# Development and application of a thin‐film molecularly imprinted polymer for the measurement of mycophenolic acid in human plasma

**DOI:** 10.1002/jcla.24864

**Published:** 2023-04-09

**Authors:** Evan Langille, Christina S. Bottaro

**Affiliations:** ^1^ Department of Chemistry Memorial University of Newfoundland St. John's Newfoundland and Labrador Canada

**Keywords:** human plasma assays, molecularly imprinted polymer, mycophenolic acid, tandem mass spectrometry, therapeutic drug monitoring, thin‐film microextraction

## Abstract

**Background:**

Mycophenolic acid (MPA) is used to suppress the immune response following organ transplantation; however, complex pharmacokinetic behavior and a large interpersonal variability necessitate therapeutic drug monitoring. To overcome the limitations of current sample preparation techniques, we present a novel thin‐film molecularly imprinted polymer (TF‐MIP) extraction device as part of a simple, sensitive, and fast method for analysis of MPA from human plasma.

**Methods:**

Mycophenolic acid is extracted from plasma using a tailor‐made TF‐MIP that is subsequently desorbed into an organic solvent system compatible with mass spectrometry. The MIP yielded higher recovery of MPA relative to a corresponding non‐imprinted polymer. The method allows for the determination of MPA in 45 min including analysis time and can be scaled for high throughput to process as many as 96 samples per hour.

**Results:**

The method gave an LOD of 0.3 ng mL^−1^ and was linear from 5 to 250 ng mL^−1^. Patient plasma samples (35 μL) were diluted using charcoal‐stripped pooled plasma to a final extraction volume of 700 μL; when MPA in patient plasma is high, this ratio can easily be adjusted to ensure samples are within the method linear range. Intra‐ and inter‐day variability were 13.8% and 4.3% (at 15 ng mL^−1^) and 13.5% and 11.0% (at 85 ng mL^−1^), respectively (*n* = 3); inter‐device variability was 9.6% (*n* = 10).

**Conclusions:**

Low inter‐device variability makes these devices suitable for single use in a clinical setting, and the fast and robust method is suitable for therapeutic drug monitoring, where throughput and time‐to‐result are critical.

## Graphical abstract

We present a novel thin‐film molecularly imprinted polymer (TF‐MIP) extraction device as part of a simple, sensitive, and fast LC–MS method for analysis of mycophenolic acid from human plasma.

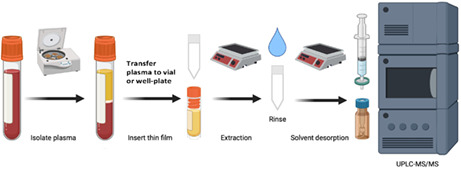

## 1 INTRODUCTION

Mycophenolic acid (MPA) is a small‐molecule pharmaceutical used as an immunosuppressant during stem cell^1^ and organ transplantation,^2^ most commonly administered after kidney transplantation as an antirejection agent. MPA acts by inhibiting inosine monophosphate dehydrogenase preventing synthesis of guanosine which in turn quells production of DNA and proliferation of T‐ and B‐lymphocytes.^3^ Since 1995, MPA has been widely adopted as the antirejection drug of choice for organ transplants,^2^ however, pharmacokinetic and pharmacodynamic variabilities for individuals and between patients continue to present challenges for optimal dosing.^4^ The observed variability can be attributed to a number of factors, including drug interactions, kidney and liver function, and disease status.^5^ The pharmacokinetics are complex; enterohepatic recirculation leads to serum concentration increases for 8–12 h after administration, followed by a rapid decrease in concentration as the drug is metabolized.^6^ Over the course of recovery from renal transplant, the bioavailability of MPA steadily increases as renal function is reestablished.^7^ The plasma concentration in the early stages of recovery can vary in an individual by as much as a factor of 4, leading to dosing challenges.^8^, ^9^ Side effects from high doses of MPA, that can be mitigated by reducing the dosage to the minimum effective concentration are abdominal pain, diarrhea, and nausea, among others.^8^, ^10^ Additionally, longer term overdosage of MPA can lead to several physiological and hematologic conditions, including the possibility of opportunistic pathogen and viral infections as well as significant damage to both the renal system and heart.^11^, ^12^ Therapeutic drug monitoring (TDM) of MPA has previously been employed when graft deterioration and compliance issues are a concern, however, logistical and method limitations are barriers to widespread TDM.^13^ Nevertheless, TDM for MPA has been previously reported using LC–MS^14^, ^15^, ^16^, ^17^, ^18^ using common sample preparation methods, including protein precipitation,^15^, ^16^, ^17^ solid phase extraction,^15^, ^19^ on‐line microdialysis,^18^ or ultrafiltration.^16^, ^17^ These approaches are largely manual, time consuming, and often require large volumes of plasma. Alternative microextraction methods are promising for TDM.^20^ One previous study reported a carbowax/templates resin SPME method with HPLC‐UV detection for the measurement of MPA in human serum with estimated LOD in plasma of 50 ng mL^−1^.^21^

Of the microextraction methods, porous thin‐film microextraction (TFME) has been employed most often for environmental samples,^22^, ^23^ with additional sensitivity and specificity imparted through incorporation of molecular imprinted polymers (MIPs).^24^, ^25^, ^26^ Recently, this approach has been extended to biological samples.^27^ By introducing molecular imprinting into polymers used in TFME (MIP‐TFME), we can add another mode of selectivity to polymeric sorbents.^28^, ^29^ MIPs are prepared by polymerization in the presence of a template molecule with functionality and shape similar to the analyte to form selective cavities in the polymer that are conserved once the template is removed. This selectivity allows for development of highly efficient extraction materials suitable for use in TDM.

Herein, a new method based on a TF‐MIP device is presented for the extraction and analysis of mycophenolic acid in human plasma. The process is time and resource efficient and capable of accurately quantifying MPA small volumes of plasma (e.g., 35 μL).

## 2 MATERIALS AND METHODS

All chemicals and reagents were purchased from Sigma‐Aldrich (Oakville, ON, Canada), were of reagent grade or higher, and were used without further purification unless otherwise noted. LC–MS solvents were purchased from Fisher Scientific (Whitby, ON, Canada). Ultrapure water was made in‐house using a Millipore Milli‐Q water system (resistivity ≥ 18.2 MΩ cm^−1^). Both ethylene glycol dimethacrylate (EGDMA) and 4‐vinylpyridine (4‐VP) were passed through gravity columns of basic aluminum oxide, Brockmann‐type I, 50–200 μm, and 60 Å to remove polymerization inhibitors; purified products were used within 3 h of purification. A pH 3.0 phosphate buffer was prepared according to the European Pharmacopeia 5.0. In short, 12.0 g anhydrous sodium dihydrogen phosphate was dissolved in 700 mL ultrapure water. The pH was adjusted by dropwise addition with stirring of 10% v/v phosphoric acid in water to a final pH of 3.0. The solution was finally diluted to a final volume of 1.0 L and the pH was checked again to confirm the final pH of 3.0 was maintained.

### 2.1 Instrumentation and operating conditions

The separation and quantification of mycophenolic acid was performed using an Acquity ultra performance liquid chromatography (UPLC) and a Xevo TQ‐S triple quadrupole mass spectrometer (Waters Corporation, Milford, Massachusetts, USA) operated in positive ionization mode equipped with an electrospray ionization (ESI) source. Chromatographic separations were carried out using an Acquity BEH‐C_18_ column (2.1 × 50 mm, 1.7 μm) maintained at 30.0 °C. Isocratic elution consisted of 40% water and 60% acetonitrile both with 0.1% formic acid at a flow rate of 0.5 mL min^−1^. The runtime of the method was 1.8 min and the retention time of MPA was 1.13 ± 0.02 min. The mass spectrometer was operated in multiple reaction monitoring (MRM) mode monitoring two transitions (Table S1) which were identified by infusion of a 50 ng mL^−1^ solution of MPA into the mass spectrometer using the IntelliStart software (Waters Corp.) and confirmed using previously reported literature.^30^ Extracted samples were stored in polypropylene vials (700 μL) at 4°C in the autosampler (SM‐FTN, Waters Corp.) prior to analysis.

### 2.2 Preparation of MIP extraction devices

Extraction devices were prepared by drop‐casting an aliquot of an optimized mixture of prepolymer complex made with 5 mmol (905 μL) EGDMA (cross linker), 1 mmol (106 μL) 4‐VP (functional monomer), 0.25 mmol (108 mg) mycophenolate mofetil (template), 16 mg 2,2‐dimethyoxy‐2‐phenylacetophenone (initiator), 130 μL acetonitrile (porogen), and 1300 μL 1‐octanol (porogen). The prepolymer solution was sonicated to dissolve the components and to degas the mixture. The solution was prepared fresh each time films were to be made. A 3.0 μL aliquot of the solution was drop‐casted between a stainless steel blade (0.5 × 2 cm) and a microscope cover slide, and then exposed to UV light (254 nm) for 20 min to induce polymerization as previously reported.^27^, ^31^ The cover slides were gently removed from the resulting films, which were subsequently washed with methanol until no template could be detected in a blank extraction using a subset of devices from each batch. Methanol, a protic solvent, disrupts hydrogen bonding between the template molecule and the polymer, allowing for template removal.

### 2.3 MIP extraction of plasma

Plasma pH was adjusted by adding 0.1 M phosphate buffer pH 3.0 to a final volume of 30% v/v. Conical vials (700 μL, polypropylene) were used for extraction of MPA from plasma. A total quantity of 700 μL of pH adjusted plasma (blank, patient samples, or spiked for method development) was added to the conical vial, followed by the MIP‐TFME device. Agitation to assist extraction was applied using a multi‐tube vortex at 1000 rpm. After a 30 min extraction, the MIP thin film was washed with 5.0 mL of ultrapure water to remove residual sample matrix components. The MPA was desorbed from the MIP devices using 700 μL of 90% acetonitrile, 9.9% water, and 0.1% formic acid in a conical vial vortex mixed at 1000 rpm for 2 min. Following desorption, MIP thin films were removed from the vial and the solution was syringe filtered (0.20 μm, 4 mm, polyethersulfone) before analysis by LC–MS/MS.

### 2.4 Plasma samples

Plasma from three patients who were prescribed MPA was purchased from BioIVT (Hicksville, NY, USA). All samples were provided as 3.5 mL isolated plasma which was shipped at −78°C on dry ice, stored at −20°C until use, and thawed at 4°C for 1 h prior to use. Patient 1 was a male (age: 65+) diagnosed with myelofibrosis, type 2 diabetes, and hypothyroidism. Patient 1 was prescribed 500 mg mycophenolate mofetil *qd*. Patient 2 was a male (age: 50+) diagnosed with end‐stage renal disease. Patient 2 was prescribed 180 mg mycophenolate sodium *qd*. Patient 3 was a female (45+) diagnosed with end‐stage renal disease, hyperparathyroidism, vitamin D deficiency, hypertension, and glomerulonephritis. Patient 3 was prescribed 360 mg mycophenolate sodium *qd*. Additional patient information is available in Table S2 of Supplementary Information.

## 3 RESULTS

### 3.1 Formulation development

**FIGURE 1:**
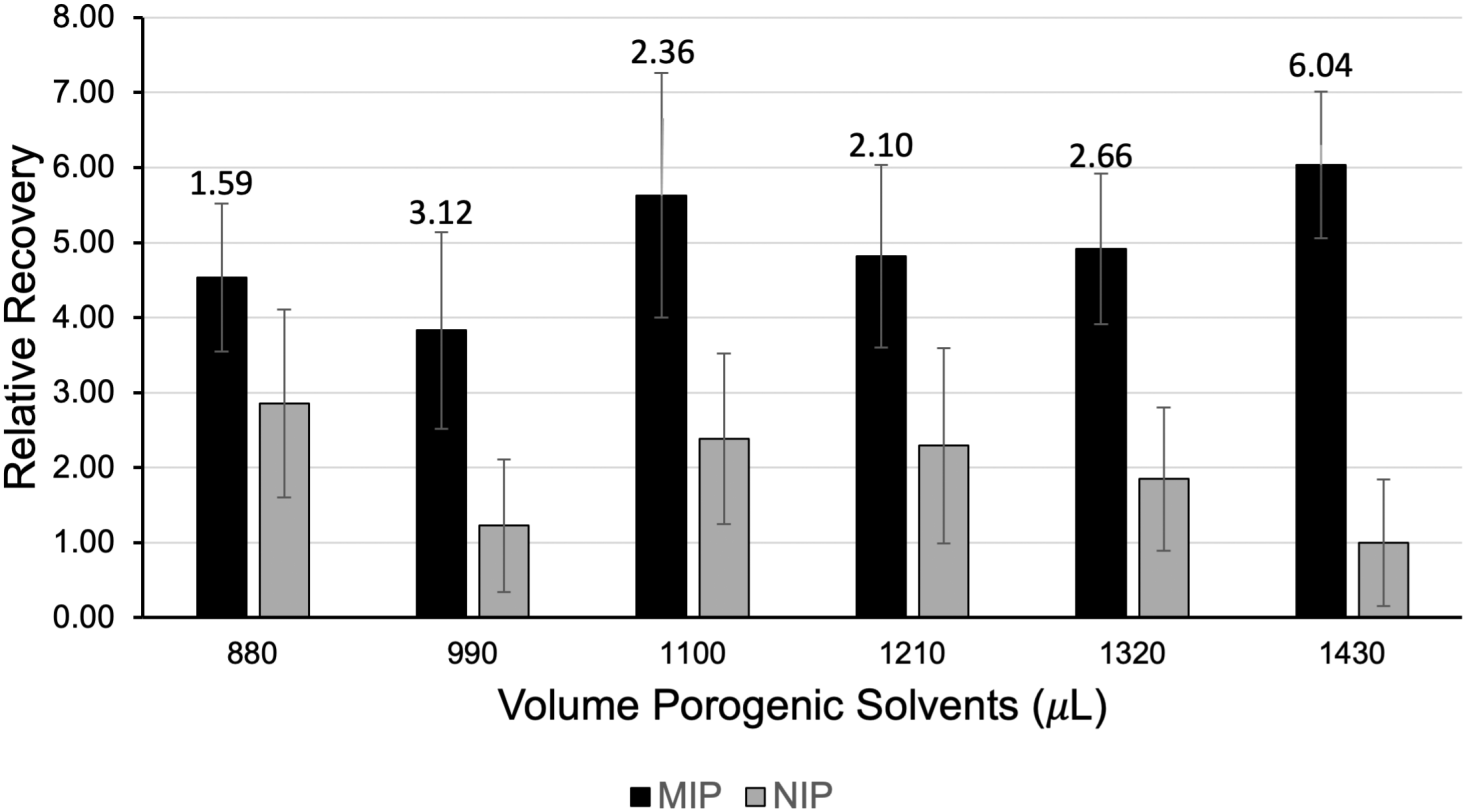
Relationship between porogen volume, relative recovery of MIP and NIP extractions of 50 ng mL^−1^ mycophenolic acid in plasma, and imprinting factor of imprinted polymers as compared to non‐imprinted polymers. Imprinting factors are shown as data labels above the MIP bars for the respective MIP/NIP pair. Data are normalized to the lowest performing NIP (1430 μL). Error bars ± SD (*n* = 3).

Mycophenolate mofetil, a morpholinyl ethyl ester and prodrug of MPA,^32^ was selected as the template due to its similarity to MPA and commercial availability. A functional monomer that can act as a proton acceptor, 4‐VP, was selected given the acidic nature of mycophenolic acid and cross‐linked with EGDMA for its appreciable biocompatibility.^33^ The porogen (porogenic solvent) was optimized by screening several solvents including: 1‐octanol, octanoic acid, diethylene glycol, diethylene glycol diethyl ether, ethylene glycol, methanol, acetonitrile, 1‐butanol, 1‐pentanol, and 1,4‐pentanediol (data not shown). The optimal solvent in terms of film stability was 1‐octanol. This produced a film with excellent mechanical stability, which is needed to limit polymer erosion during rapid agitation and physical manipulation of the MIP‐TFME devices in the extraction process. The porogen solvent system was modified with 10% v/v acetonitrile required to solubilize mycophenolate mofetil. Due to the crucial role in formation of a porous sorbent, porogen loading is one of the most important factors to optimize when developing thin‐film MIPs. Experiments to determine the optimal volume of porogen in the prepolymer complex solution (Figure 1) demonstrate how subtle changes in porogen loading can lead to significant changes in adsorption behavior. We have reported similar phenomena for other MIPs.^31^ The highest total MPA recovery was obtained with the formula with 1430 μL, which corresponds to the most diluted concentration of prepolymer components in the series but results in a film with superior mechanical stability as compared to less dilute prepolymerization candidates. Increasing the amount of porogen relative to the polymer components led to a dramatic decrease in recovery for the non‐imprinted polymer and a general increase in variability between devices. This is likely the result of a polymer with larger pores and lower surface area due to the increased proportion of porogen. The variability improves with increased volume of porogenic solvent for the MIP. Ultimately, this led us to select the 1430 μL formula, which also revealed the greatest imprinting factor (IF 6.04) and MPA recovery. The total volume of porogen in the final formula is 1300 μL 1‐octanol and 130 μL acetonitrile.

### 3.2 Optimization of the desorption conditions

**FIGURE 2:**
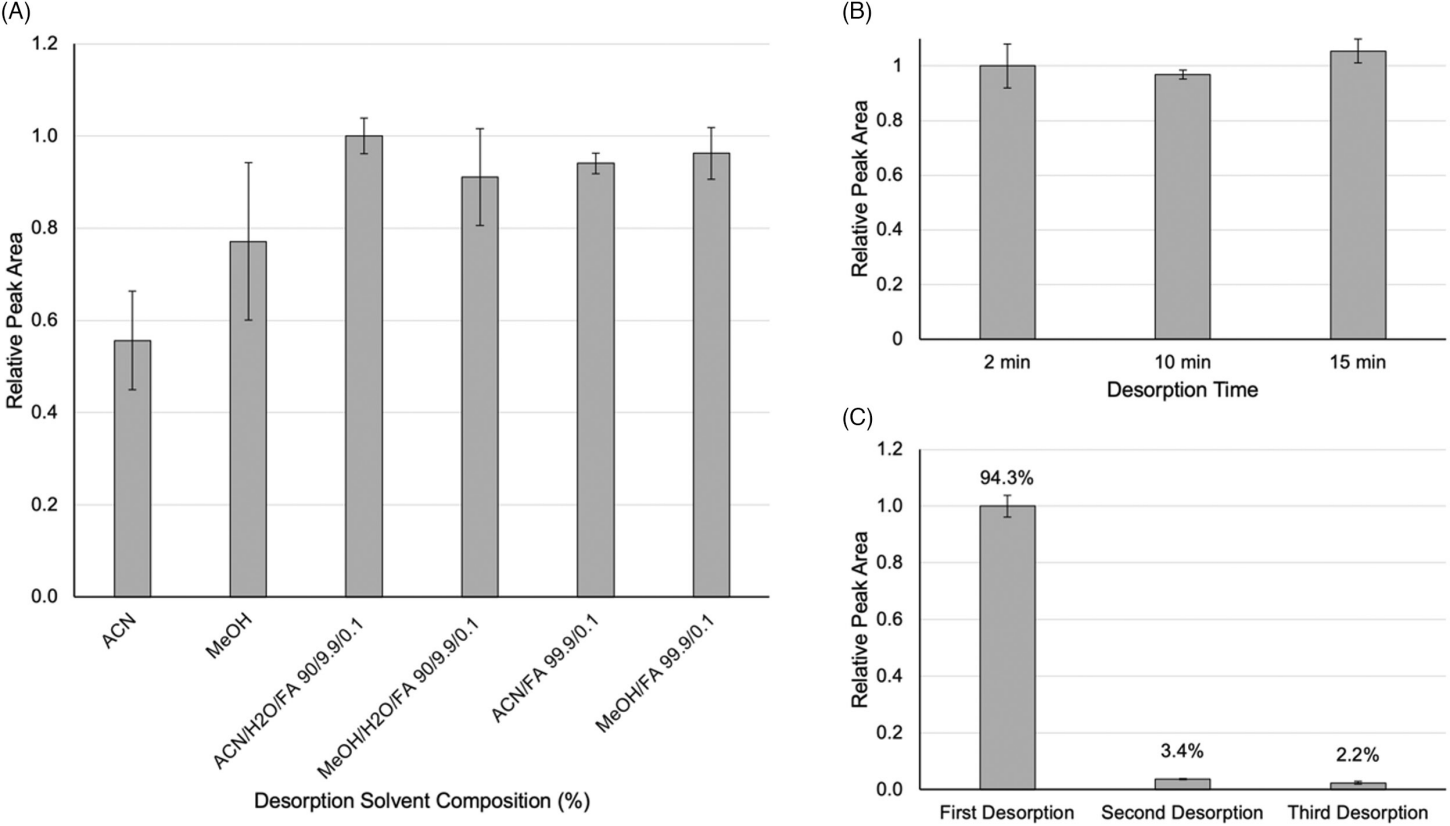
(A) Comparison of relative recovery of MIP extraction of 50 ng mL^−1^ mycophenolic acid in plasma with different desorption solvents. The relative peak area is relative to the final selected conditions (ACN/H_2_O/FA 90/9.9/0.1). (B) Comparison of desorption time of MIP extraction of 50 ng mL^−1^ mycophenolic acid in plasma with 90% acetonitrile, 9.9% water, and 0.1% formic acid. Relative peak area is relative to the final selected desorption time (2 min). (C) Sequential 2 min desorption of MPA extracted by MIP‐TFME from 50 ng mL^−1^ mycophenolic acid in plasma. Desorption solvent: 90:10 acetonitrile: 0.1% aqueous formic acid. Error bars ±SD (*n* = 3).

Quantitative desorption from the sorbent is required for a reliable analytical method. Various solvent systems and desorption times were studied to determine the optimum desorption conditions. Acetonitrile, methanol, water, and formic acid in various mixtures were chosen as potential candidates for the desorption solvent, due to their compatibility with the chromatographic separation (Figure 2A). From this data, it can be observed that the highest desorbed recovery was achieved using a solvent system consisting of 90% acetonitrile, 9.9% water, and 0.1% formic acid. Another factor in selecting acetonitrile, as opposed to methanol, is that it shows better compatibility with the LC solvent system, yielding narrower, more symmetrical peaks in chromatography.

We then sought to optimize desorption time to determine the minimal time required to extract the majority of the MPA from the film. We tested 2, 10 and 15 min desorption times (Figure 2B). We selected these times based on initial screening that showed that the desorption process with selected solvent was quite fast. We observed no significant difference between desorption times tested where both 10 and 15 min desorption intervals were yielding the same recovery compared to a 2 min desorption. The variability in standard deviation (SD) is since for each time tested, we used three individual devices, thus there is expected inter‐device variability contributing to the presented error. In conclusion, a single, 2 min desorption with 90% acetonitrile, 9.9% water, and 0.1% formic acid is used to quantitatively desorb the extracted MPA from the thin films.

We then investigated if a single 2 min desorption could recover the majority of the extracted mass (Figure 2C). Nearly 95% of the extracted mass is recovered in the first desorption while 3.4% and 2.2% are recovered during second and third desorption steps, respectively.

### 3.3 Optimization of extraction conditions

**FIGURE 3:**
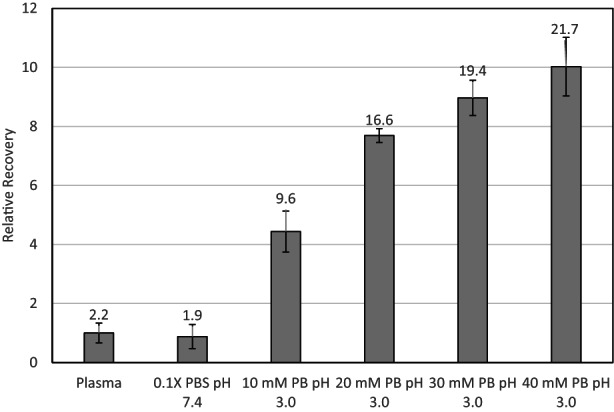
Comparison of relative recovery of MIP extraction of 50 ng mL^−1^ mycophenolic acid in plasma, and with addition of phosphate buffer solution (PBS). 0.1× PBS pH 7.4 contains the following: 13.7 mM NaCl, 0.27 mM KCl, 1.0 mM Na_2_HPO_4_, and 0.18 mM KH_2_PO_4_. Relative recovery to unadjusted plasma. Data labels are percentage recovery, error bars ±SD (*n* = 3).

The pH of the sample during extraction can have a significant impact on the extraction efficiency mainly due to the ionization of labile protons (pK_a_). As MPA is neutral in its protonated form (pH < pK_a_), reducing the plasma pH will convert more MPA to its neutral form, which is favored for adsorption to the thin film. We compared unadjusted plasma, plasma supplemented with 0.1× PBS pH 7.4 (10% v/v), and plasma with varying amounts (10–40% v/v) of a 0.1 M pH 3.0 phosphate buffer (Figure 3). When increasing amounts of pH 3.0 phosphate buffer are added to the plasma, we saw marked increase in total MPA recovery, whereas recovery was slightly reduced with addition of PBS (pH 7.4). The increased recovery due to sample acidification was as high as 10× relative to unadjusted samples, which demonstrates the need for pH adjustment in the plasma samples as a pretreatment before extraction. Although addition of 40 mM concentration led to the highest apparent recovery, the increased inter‐sample variability is a demerit and meant that the recovery was not statistically different than adjustment with 30 mM PB pH 3.0. A final concentration of 30 mM PB pH 3.0 was selected with 19.4% recovery relative to 2.2% for the unadjusted samples. The dilution of plasma samples is taken into account mathematically when calculating the plasma concentration. As this is a non‐exhaustive equilibrium‐based extraction regime, we do not expect 100% recovery, and thus the obtained percentage recoveries presented as data labels in Figure 3 are sufficient to obtain the required clinical lower limits of quantification for the method.

### 3.4 Optimization of extraction time and extraction linearity

**FIGURE 4:**
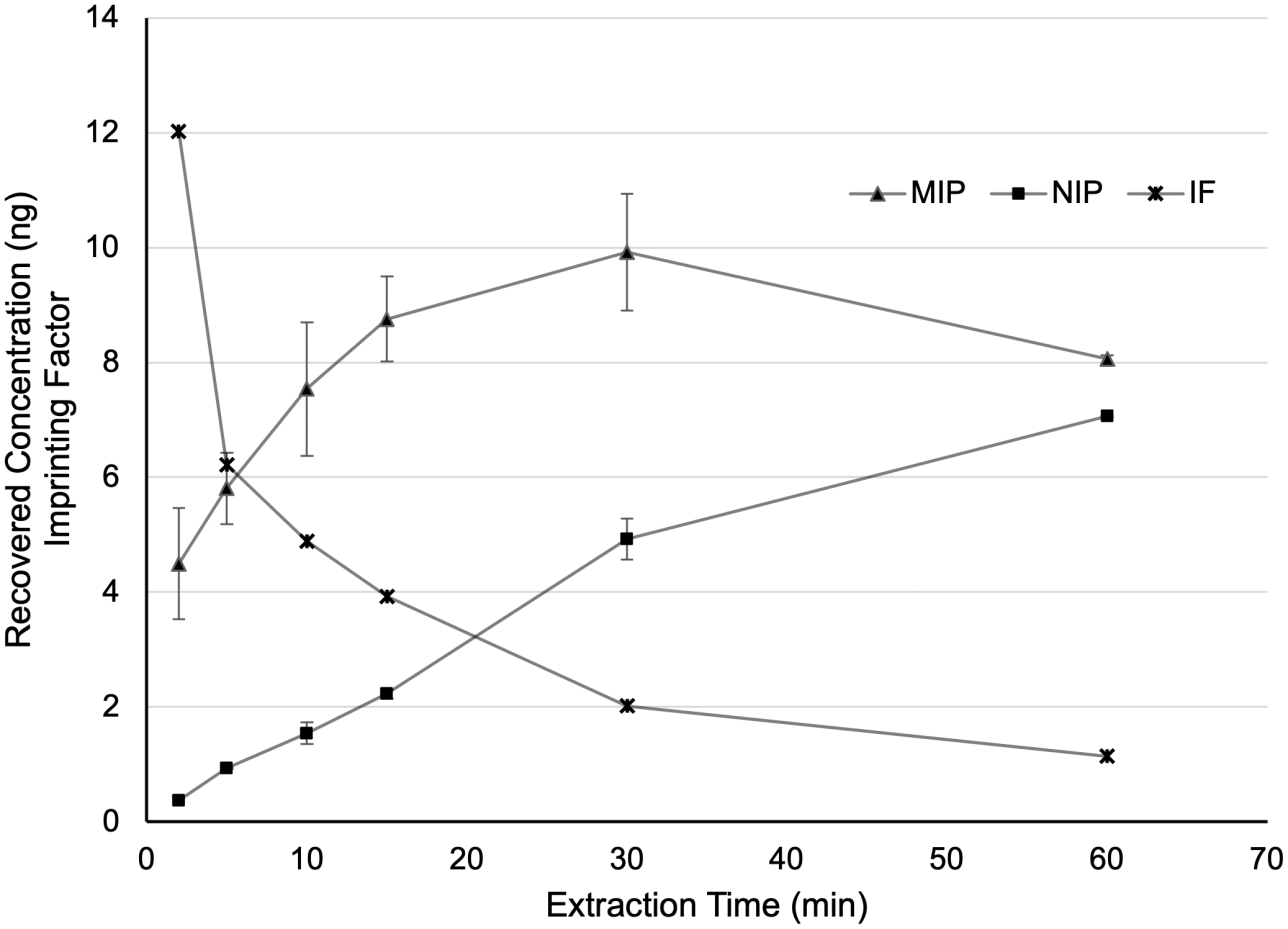
Time profile of MIP and NIP extractions of 50 ng mL^−1^ mycophenolic acid from plasma, and the corresponding imprinting factor at each time point. IF represents the imprinting factor of the molecularly imprinted polymer as compared to the non‐imprinted polymer at each time point. Error bars ±SD (*n* = 3).

To determine optimal extraction time, an extraction time profile was generated which compared the MIP extractions, NIP extractions, and imprinting factor at varying time points (Figure 4). We observe a significant imprinting effect from the MIP at early time points, with the NIP lagging in initial extraction rate. From the observed trends, 30 min extractions were selected as they had relatively high recovery but were still rapid enough not to be logistically limiting in the laboratory when processing many samples in parallel. This time point also appears to be nearly at equilibrium for the MIP, but not the NIP, and gives an imprinting factor of 2 at this time.

**FIGURE 5:**
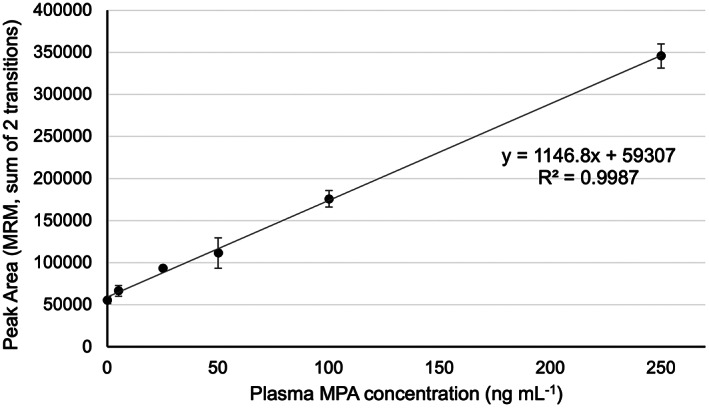
Extraction calibration curve of mycophenolic acid in plasma. Error bars ±SD (*n* = 3).

Using the 30 min extraction time, an extraction calibration curve was generated from spiked, pooled plasma (Figure 5). The extraction of MPA from plasma using this device is linear from 5–250 ng mL^−1^. The obtained linear range is relevant to clinical samples as the target concentration of free MPA in the plasma is expected to be approximately 50 ng mL^−1^.^34^

### 3.5 Analytical performance thin‐film MIPs for determination of MPA in plasma

**FIGURE 6:**
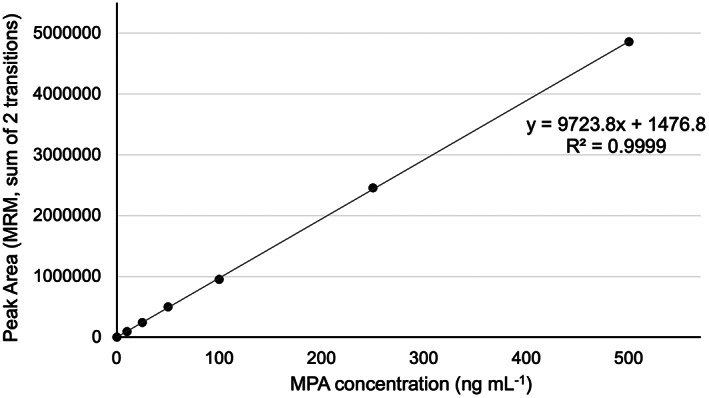
External calibration curve of mycophenolic acid from 1 to 500 ng mL^−1^. Error bars ±SD and are too small to be observed on this scale. The %RSD ranged from 0.4% to 1.69% with an average RSD per point of 0.77% (*n* = 3).

The sum of the peak areas for two MRM transitions (321.1 → 159.0 and 321.1 → 207.0) for MPA were used for all quantitation. An external calibration curve was prepared to determine the instrumental linear range (Figure 6). We determined the instrumental response to be linear from 1–500 ng mL^−1^. This range is suitable for the extraction calibration range that has been determined for our device (Figure 5).

### 3.6 Analytical figures of merit

The figures of merit for the method are presented in Table S3. The inter‐device variability was slightly less than 10% (*n* = 10). This indicates that the devices could be used as a disposable device with high repeatability associated with manufacturing of the coatings. The method working range is from 5 to 250 ng mL^−1^ and was linear across all concentrations studied. The dose of free MPA observed in patients is expected to fall within both the method and instrumental (1–500 ng mL^−1^) linear ranges. The expected concentration of free MPA in the plasma ranges from 5 to 270 ng mL^−1^,^5^, ^30^ while the total MPA in plasma ranges from 1000 to 3500 ng mL^−1^.^1^ As we did not incorporate a hydrolysis step into the method, we expect to be measuring free MPA.

### 3.7 Analysis of patient samples

As organ transplantation is not conducted in the province of Newfoundland and Labrador, we did not have access to fresh patient specimens undergoing treatment with an MPA‐based regimen. Alternatively, we sourced donated plasma from the United States of patients who are undergoing MPA treatment. The plasma was thawed to be aliquoted following shipment to us, in addition to several freeze–thaw cycles from unplanned power outages on campus and the COVID‐19 pandemic lockdowns. Upon return to the laboratory, we attempted to acquire new samples, however, the pandemic situation in the United States resulted in no sample availability due to closure of collection sites.

We suspected the samples may have elevated free MPA concentrations as repeated freeze–thaw cycles and elevated temperatures will cause bound MPA to degrade back into the unbound form. As anticipated, patient samples showed very high concentrations of MPA in the plasma outside of the calibration range of the method. To accurately quantify MPA in the treated patient samples, a 1:20 dilution in twice charcoal‐stripped pooled plasma was used as a diluent for extraction before pH adjustment as previously described. By using charcoal‐stripped plasma for dilution of the samples, a consistent amount of matrix was present in the samples allowing for variable dilutions based on patient dosage, while maintaining the complexity of the sample with respect to potential interference by endogenous compounds and maintaining consistency in the physicochemical properties of the sample. The method could also be modified to incorporate dilution with standard buffer systems, which will not diminish device performance. In the case of our plasma samples, only 35 μL was used for each extraction representing a 20× dilution. This allows for a broader range of concentrations to be measured using this method, by adjusting the volume of patient sample used, should it be necessary. However, the greatest advantage to the method is the small sample size required which allows for a reduction in the required blood draw from the patient, enabling more tests to be conducted on less blood, and thus less harm to the patient. As observed in Table 1, the measured plasma concentrations correlate well with the daily prescribed doses bearing in mind that pharmacodynamics vary dramatically between patients.

**TABLE 1:** Results of patient sample analysis for MPA.

Patient	Measured concentration plasma x20 dilution (avg ng mL^−1^, *n* = 3)	RSD (%)	Plasma concentration (mg mL^−1^)	Daily prescribed dose (mg, as MPA)
1	196 ± 20	10	3.92	369
2	49.2 ± 1.5	3	0.984	168
3	190 ± 19	10	3.79	337

*Note*: Plasma concentrations correlate with daily prescribed dosage. Samples were quantified using the extraction calibration curve (Figure 7) generated by spiked pooled plasma.

**FIGURE 7:**
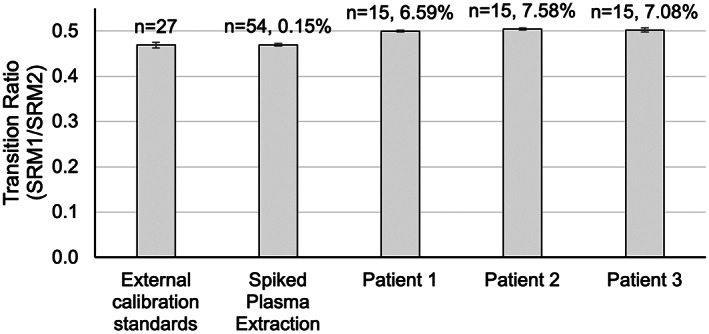
Transition ratio monitoring of two channels in calibration standards, spiked pooled plasma and patient samples. For each column, *n* = the number of injections for each sample is presented with percentage difference between the response for the samples relative to calibration standards. Error bars are ±SD for *n* measurements.

The transition ratios for MPA were tabulated for all types of experiments as a simple assessment of matrix effects. Ratio variability is within acceptable ranges as defined by the Clinical Laboratory Standards Institute (CLSI) C50‐A guidance documentation.^35^ The maximum allowable tolerance for a second transition is ≤25% for a transition that is 20%–50% of the base peak response. For 126 measurements, the average transition ratio was stable at 0.489 ± 0.018 (Figure 7). This small variability between calibration standards, matrix, and patient samples indicates that spectral matrix effects are minimal. Blank matrix extraction of plasma gives signal far below the LOD of the method, indicating that the MIP extraction was successful in removing potential chromatographic interferents, as compared to plasma that is directly injected in which large, interfering peaks can be observed. The overall variability of 3.7% for a transition at ~49% of the base peak, with the authentic samples giving less than 8%, is well within prescribed limits.

## 4 DISCUSSION

A MIP thin‐film device was developed to rapidly extract MPA from human plasma. The MIP yielded a higher recovery of the drug compared to the analogous non‐imprinted polymer (NIP). The method was optimized using pooled blank human plasma. Analysis of an extraction time profile showed 30 min provides sufficient recovery to meet detection limits required for the MPA therapeutic clinical range. The optimized desorption solvent system (90% acetonitrile, 9.9% water, and 0.1% formic acid) provided high desorption efficiency for MPA in 2 min. The full method, including sample preparation and UPLC–MS analysis, can be completed in 45 min. Use of equipment for multiplexed sample processing (e.g., a multi‐position vortex mixer), allows a single technician to process more than 96 samples per h.

The LOD and LOQ are 0.3 and 1.0 ng mL^−1^, respectively, with a linear range from 5 to 250 ng mL^−1^. The intra‐ and inter‐day variability was determined to be 13.8% and 4.3% (15 ng mL^−1^) and 13.5% and 11.0% (85 ng mL^−1^), respectively (*n* = 3). The inter‐device variability was 9.6% (*n* = 10). The low inter‐device variability makes these devices suitable for single use in a clinical setting. Due to complexities with the acquisition of patient plasma, the samples tested were above the linear range of the method. The volume of patient plasma was reduced to 35 μL using the method for the samples to place concentrations within the linear range of the method. We expect that with fresh patient plasma, we would be able to effectively measure free MPA in the linear range of the method. Dilution of the patient samples (20×) was completed with charcoal‐stripped pooled plasma which allowed for less volume requirement from patients and standardization of the amount of matrix independent of treated plasma input. This demonstrates that the method can be adapted for limited plasma volumes with minimal effect on performance, demonstrating the highly flexible nature of MIP thin‐film extraction devices for both clinical applications and TDM. Plasma was obtained and analyzed from patients prescribed MPA. The amount of MPA in the samples ranged from 984 to 3924 ng mL^−1^ with an average RSD of 7.7% (*n* = 3). As demonstrated by the required dilution of the degraded plasma, this method could be easily modified to include a hydrolysis step to measure total MPA, if desired. The main modifications to the protocol would be hydrolysis followed by dilution of the sample, approximately 20×.

The MIP devices reported provide an efficient method for extraction of MPA from plasma with sampling to result in 45 min. As the extraction system is easily multiplexed, throughput is high. The MIP films demonstrate a high level of reproducibility and affinity for the analyte assisted through molecular imprinting. This novel method and device could be used for TDM of MPA in a clinical setting where throughput and time‐to‐result are critical. This approach can easily meet sensitivity requirements while using small volumes of plasma as demonstrated by 35 and 700 μL volumes used in this study. Since the cost‐effective single‐use devices can be made quickly and efficiently, they can be used to increase throughput in clinical laboratories and are adaptable for use in microplate preparation systems.

## AUTHOR CONTRIBUTIONS

All authors have accepted responsibility for the entire content of this manuscript and approved its submission. E.L. and C.B. wrote the article; CB provided funding, laboratory space, and equipment; E.L. designed the research; performed the experiments, and analyzed the data.

## FUNDING INFORMATION

This work was supported by Atlantic Innovation Fund (AIF) Project# 781–18,607‐208,053 from Atlantic Canada Opportunities Agency (ACOA), Newfoundland and Labrador Department of Tourism, Culture, Industry and Innovation, the Natural Sciences and Engineering Research Council of Canada (NSERC), Discovery Grant #RGPIN‐2015‐06367, and the Department of Chemistry and School of Graduate Studies (SGS) at Memorial University of Newfoundland (MUN).

## CONFLICT OF INTEREST STATEMENT

Authors state no conflict of interest.

## Supporting information


Appendix S1

## Data Availability

All data analyzed during this study are included in this article and its supplementary information files. Raw data may be made available upon request.
