# Supplementary material for: Development and application of a thin‐film molecularly imprinted polymer for the measurement of mycophenolic acid in human plasma
Source: J Clin Lab Anal. 2023 Apr 9;37(6):e24864. doi: 10.1002/jcla.24864 (PMC10156102; doi:10.1002/jcla.24864)
Supplement: Supplementary file 1 — Appendix S1 [file JCLA-37-e24864-s001.docx]

**Supplementary Information**

**Table S1. MRM transitions and mass spectrometer settings.**

| **Precursor Ion (m/z)** | **Product Ion (m/z)** | **Dwell Time**  **(s)** | **Cone Voltage (V)** | **Collision energy (eV)** |
| --- | --- | --- | --- | --- |
| 321.1 | 159.0 | 0.100 | 14 | 30 |
| 321.1 | 207.0 | 0.100 | 14 | 22 |

**Table S2. Patient information**

| **Patient** | **1** | **2** | **3** |
| --- | --- | --- | --- |
| Anticoagulant | Sodium heparin | Dipotassium EDTA | Dipotassium EDTA |
| Gender | Male | Male | Female |
| Age | 68 | 52 | 48 |
| Race/Ethnicity | Caucasian | Hispanic | Other |
| Diagnosis | Myelofibrosis  Type 2 diabetes Hypothyroidism | End stage renal disease | End stage renal disease Hyperparathyroidism  Vitamin D deficiency  Hypertension  Glomerulonephritis |
| Medications | Actos 30 mg  Aricept 5 mg  Aspirin 81 mg  **Cellcept 500 mg**  **(mycophenolate mofetil)**  Lyrica 50 mg  Metformin 500 mg  Namenda 5 mg  Plavix 75 mg  Prednisone 10 mg  Ramipril 5 mg  Synthroid 125 μg | Amlodipine 10 mg  Atorvastatin 40 mg  Epogen 300 units  Humalog 8 units  Lantus 50 units  **Myfortic 180 mg**  **(mycophenolate sodium)**  Prograf 1 mg | Lisinopril 5 mg  Lovastatin 40 mg  Furosemide 40 mg  **Myfortic 360 mg**  **(mycophenolate sodium)**  Tacrolimus 10 mg  Norvasc 10 mg  Omeprazole 20 mg  Prednisone 5 mg  Prograf 1 mg  Rocaltrol 0.25 μg |

Table S3: Analytical figures of merit for thin film MIP devices for the analysis of mycophenolic acid from human plasma.

| Instrumental linear range | 1-500 ng⋅mL^-1^ |
| --- | --- |
| Method linear range | 5-250 ng⋅mL^-1^ |
| Instrumental limit of detection (LOD) | 0.3 ng⋅mL^-1^ |
| Instrumental lower limit of quantification (LLOQ) | 1 ng⋅mL^-1^ |
| Intra-day variability | 15 ng⋅mL^-1^ = 13.8% (n=3)  85 ng⋅mL^-1^ = 4.3% (n=3) |
| Inter-day variability | 15 ng⋅mL^-1^ = 13.5% (n=3)  85 ng⋅mL^-1^ = 11.5% (n=3) |
| Inter-device variability | 9.6% (n=10) |


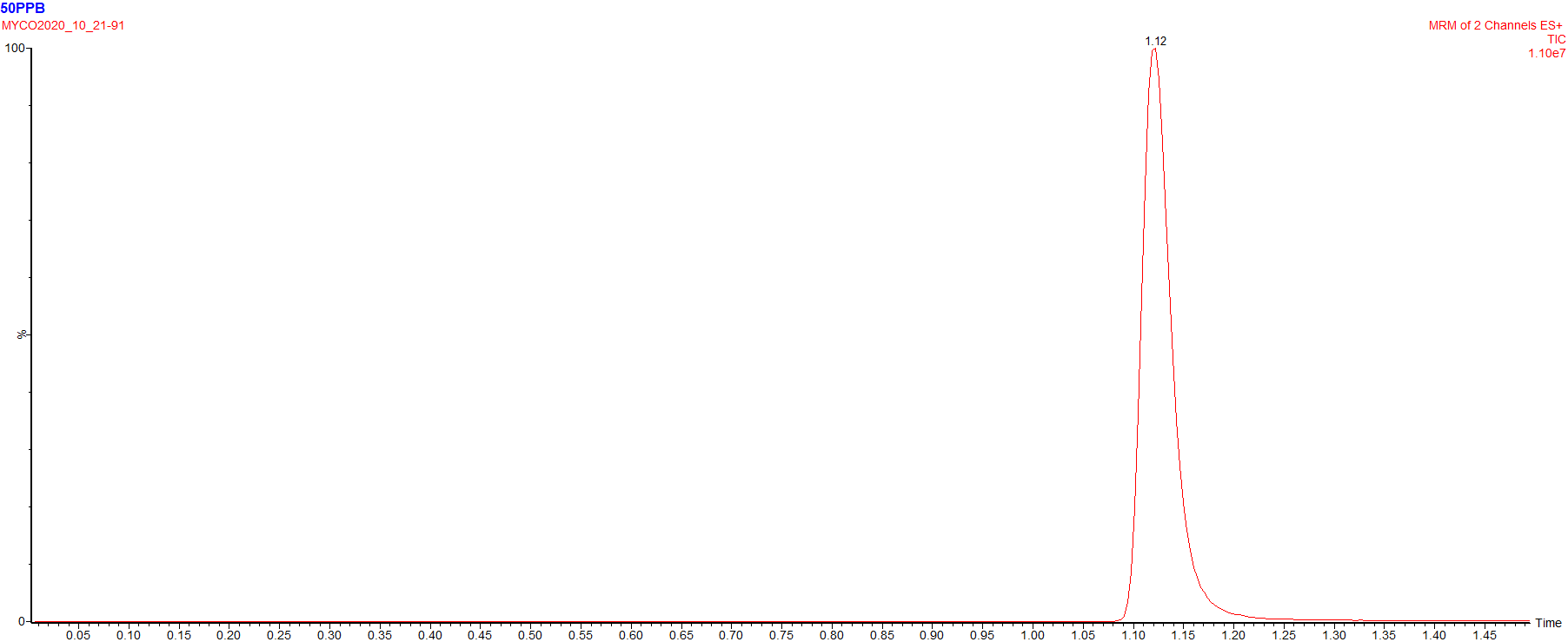


Figure S1. Example chromatogram of 50 ng⋅mL^-1^ MPA standard.


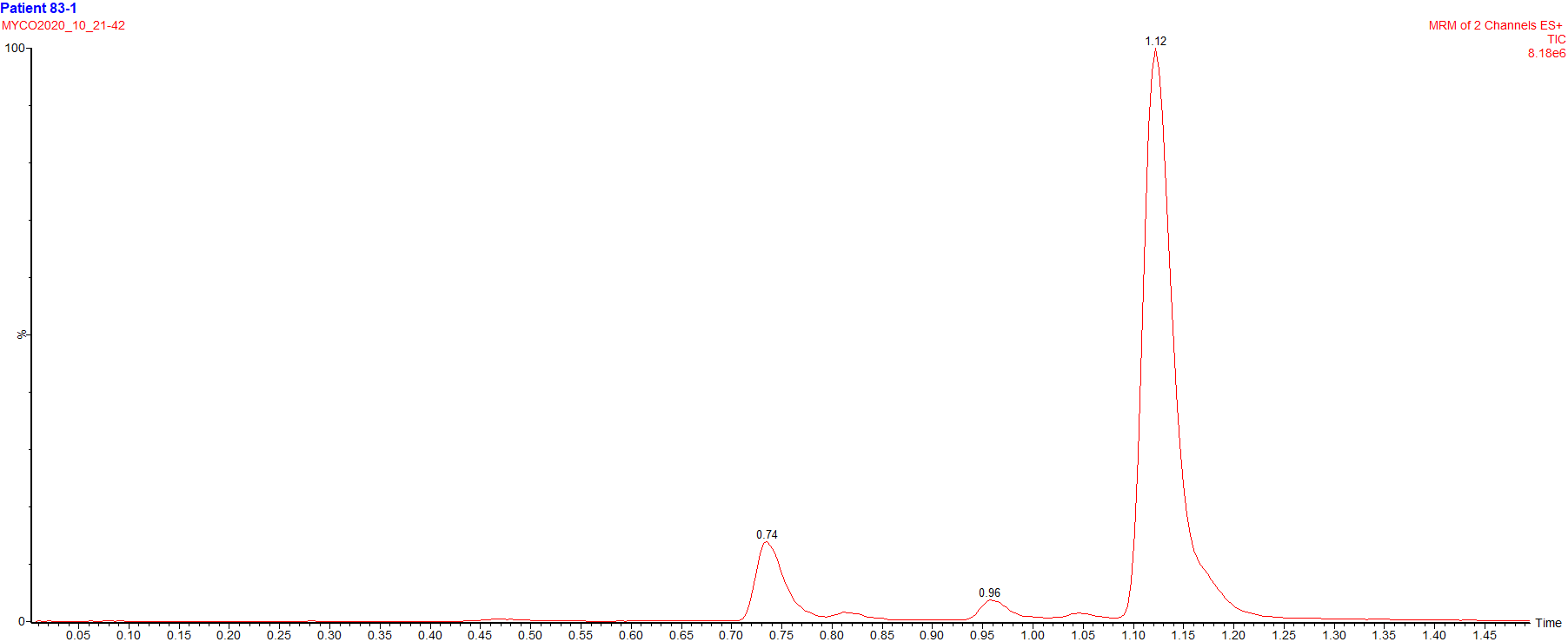


Figure S2. Example chromatogram of 20x diluted patient sample (patient 1).


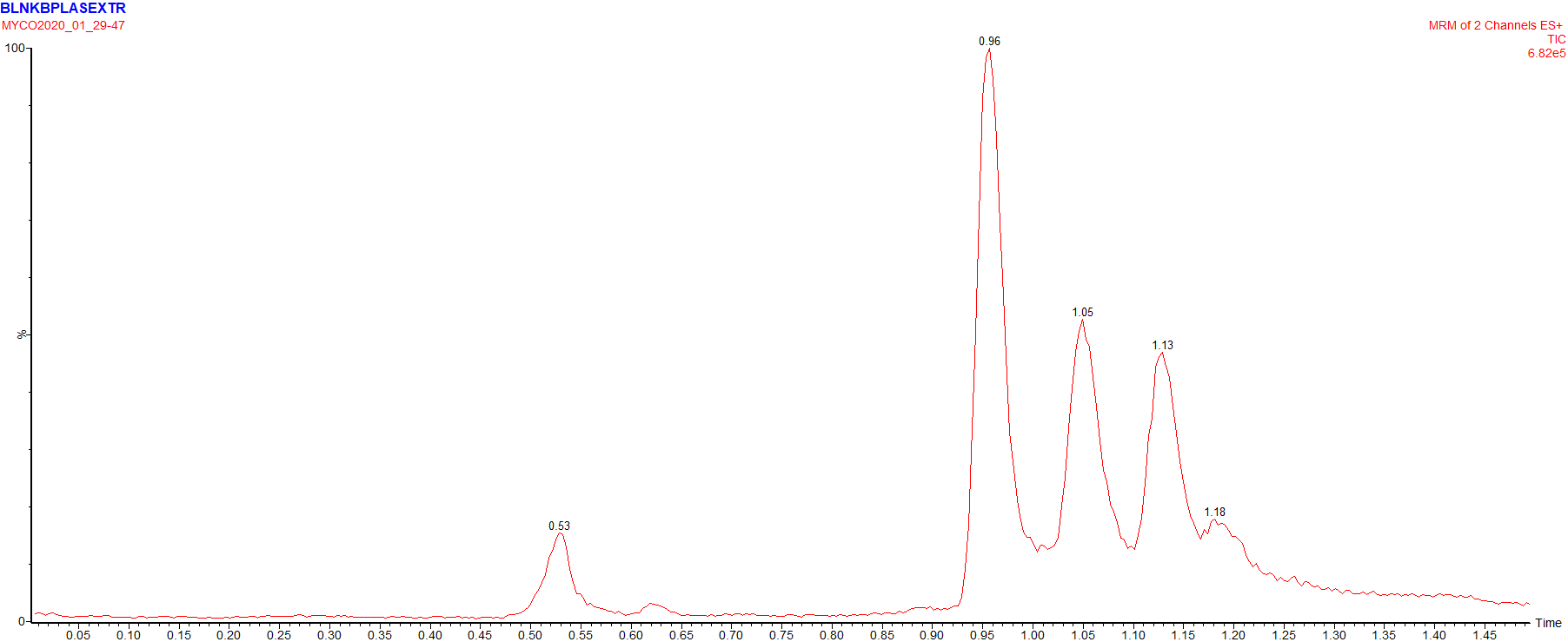


Figure S3. Example chromatogram of blank plasma extraction from pooled plasma.
